# Depression subtypes, suicidality, and healthcare costs in older adults: results from a naturalistic study

**DOI:** 10.3389/fpsyt.2025.1560719

**Published:** 2025-06-17

**Authors:** Ismael Conejero, Alejandro Porras-Segovia, Lucía Albarracín-García, María Luisa Barrigón, Jorge Lopez-Castroman, Philippe Courtet, Enrique Baca-Garcia

**Affiliations:** ^1^ Department of Psychiatry, Hospital Universitario Fundación Jiménez Díaz, Instituto de Investigación Sanitaria Fundación Jiménez Díaz, Universidad Autónoma de Madrid, Madrid, Spain; ^2^ Department of Psychiatry, CHU Nîmes, Institut de Génomique Fonctionnelle, University of Montpellier, Montpellier, France; ^3^ Departamento de Psiquiatría, Hospital Rey Juan Carlos Móstoles, Madrid, Spain; ^4^ Institute of Psychiatry and Mental Health, Hospital General Universitario Gregorio Marañón, Instituto de Investigacion Sanitaria Gregorio Maranon (IiSGM), Centro de Investigación Biomédica en Red de Salud Mental (CIBERSAM), Instituto de Salud Carlos III (ISCIII), School of Medicine, Universidad Complutense, Madrid, Spain; ^5^ Department of Psychiatry, Radiology, Public Health, Nursing and Medicine, University of Santiago de Compostela, A Coruña, Spain; ^6^ Department of Emergency Psychiatry and Acute Care, Centre Hospitalier Universitaire Montpellier, University of Montpellier, Montpellier, France; ^7^ Departamento de Psiquiatría, Universidad Autónoma de Madrid, Madrid, Spain; ^8^ Department of Psychology, Universidad Católica del Maule, Talca, Chile; ^9^ Departamento de Psiquiatría, Hospital Central de Villalba Villalba, Madrid, Spain; ^10^ Departamento de Psiquiatría, Hospital Universitario Infanta Elena Valdemoro, Madrid, Spain; ^11^ Centro de Investigación Biomédica en Red de Salud Mental (CIBERSAM), Madrid, Spain; ^12^ Department of Psychiatry, CHU Nîmes, Nîmes, France

**Keywords:** older adults, depression, suicide, healthcare costs, suicidal ideation

## Abstract

**Introduction:**

The burden of depression is increasing worldwide, particularly in older populations. While the impact of depressive disorders on suicide in later life has been clearly identified, less is known about the role of their subtypes and their costs in the elderly.

**Objectives:**

We aimed to describe the sociodemographic and clinical characteristics associated with the depression subtypes and suicidality, and their related healthcare costs in older adults receiving mental healthcare.

**Methodology:**

The study was carried out across four psychiatry departments in Madrid, Spain. Adults aged over 60 years were included if they attended the psychiatric inpatient or outpatient services and were diagnosed with Major Depressive Disorder (MDD), recurrent depressive disorder, bipolar depression, or dysthymia. Sociodemographic data and diagnoses according to the International Classification of Diseases, 10^th^ edition were obtained from electronic health records. Lifetime suicidal history, suicidal ideation and suicide attempts in the previous month were identified using the Columbia Suicide Severity Rating Scale (CSSRS). Mean healthcare costs were calculated over one year.

**Results:**

N=2868 patients were included in the analysis. Of these, 550 were assessed with the CSSR. The mean age of the sample was 70.05 years and 75.9% of the patients were women (N=2177). Of the patients assessed with the CSSRS (N=550), 83.2% (N=458) reported suicidal ideation, and 7.3% (N=40) had attempted suicide in the previous month. Psychiatric healthcare costs over the follow-up differed between the depression subtypes (Eta-squared 0.003 CI [0.000 - 0.008]; p<0.001). They were significantly higher in patients diagnosed with bipolar depression, than in those diagnosed with dysthymia (p=0.026), but did not differ from those bearing MDD (p= 0.775) or recurrent depressive disorder (p= 0.129). Recent suicide attempters had a more frequent lifetime history of suicide attempt (OR= 8.434). Suicide attempts were more frequent in individuals aged 71-80 years (OR= 3.433) or over 80 years (OR= 3.322), and in patients with recurrent depressive disorders (OR= 3.529).

**Conclusion:**

Psychiatric healthcare costs differed between depression subtypes, with a small effect. Furthermore, older age, a diagnosis of recurrent depression, and a history of suicide attempts increased the risk of suicide. Health policies should target these populations to improve mental health outcomes in the older adults.

## Introduction

The burden of depression is increasing worldwide, particularly in older populations as a result of longer life expectancy (1). The global prevalence of major depressive disorder (MDD) in the elderly ranges from 13% to 31% (2, 3), with Europe showing the second highest rates after Australia (2). Globally, depressive disorders are placed among the three leading causes of disability, along with low back pain and headache disorders (4). In parallel, suicide risk increases with age (5). The global suicide rate for people aged 50-69 years, is 16 per 100 000, but rises to 27 per 100 000 among those aged 70 and over (6). Deaths by suicide increased among older people by 8.1 per cent in the 65+ age group between 2021 and 2022 (5). The situation is equally concerning in European countries, such as Spain, where the incidence of suicide attempts among older adults has been estimated at 35 per 100 000 (7).

A recent meta-analysis examined the sociodemographic and clinical factors associated with MDD in older adults. Key factors associated with higher rates of depression in this population included female gender, advanced age, social isolation, lack of social support, and chronic health issues (3). In addition, lower levels of social activity and repeated depressive episodes may increase the risk of depression in later life (7, 8). Suicidal behavior in older adults shares common risk factors with depression including bereavement, dependency and the experience of illness (6). However, less is known regarding the distribution of these factors across unipolar and bipolar depression subtypes and their specific role in suicidal ideation or attempts in patients with depressive disorders. While the impact of depressive disorders on suicide in late life has been clearly identified (6, 9), less is known about the role of the specific depression subtypes.

In addition, depressed or suicidal older people are frail and particularly at risk of poor mid- and long-term medical outcomes (10). Therefore, it is important to target this population, which is also more likely to need healthcare resources. To this end, examining the costs associated with clinical depression subtypes and suicidal behaviors in older patients should further help to identify unmet needs in this complex population. This may also provide information to better guide large-scale health policies in depressed older adults (11).

Hence, we aimed to describe and compare the sociodemographic characteristics and clinical features associated with depression subtypes and with suicidality in older adults aged over 60 years receiving mental healthcare in hospital psychiatric inpatient or outpatient settings. We also assessed mean total, psychiatric and medical healthcare costs during follow-up according to the depression subtypes and to the occurrence of recent suicidal ideation or attempts. We hypothesized that bipolar depression, and suicidal behavior would be associated with higher levels of psychiatric comorbidities and greater healthcare costs in older age. We also hypothesized that recent suicidal behaviors in old age would be associated with a lifetime history of suicide attempts.

## Materials and methods

### Study design and patient sample

Depressed older adults aged over 60 years were recruited between 2014 and 2020 in the Departments of Psychiatry of four hospitals of the Community of Madrid, Spain (University Hospital Fundación Jiménez Díaz, University Hospital Rey Juan Carlos, General Hospital of Villalba, and University Hospital Infanta Elena).

The patients were included if they attended the psychiatric inpatient or outpatient facilities, and if they were diagnosed with MDD, recurrent depressive disorder, bipolar depression, or dysthymia. The diagnoses were coded according to the International Statistical Classification of Diseases and Related Health Problems 10th (ICD-10) criteria. In the ICD-10 classification, MDD relates with depressive episodes (F32.x) and include mild (F32.0), moderate (F32.1), severe (F32.2), or psychotic (F32.3) depressive episodes. Patients with unspecified depressive disorders (F32.9) were not included in the study. Recurrent depression (F33.x) corresponds to repeated episodes of current mild (F33.0), moderate (F33.1), severe (F33.2), or psychotic (F33.3) depressive episodes. Patients with recurrent depressive disorder unspecified (F33.9) were not included in the study. Dysthymia (F34.1) corresponds to a chronic depression of mood without criteria for diagnosing recurrent depression. Finally, bipolar depression corresponds to F31.3, F31.4 and F31.5 subcategories of bipolar affective disorders (F31.x). Regarding comorbidities, the category “organic mental disorders” (F00-F09) included Alzheimer’s dementia, delirium and other dementia; the category “substance use disorders” (F10-F19) included all the mental and behavioral disorders due to psychoactive substance use; the category “psychotic disorders” (F20-F25, F28) included schizophrenia, delusional disorders and other nonorganic psychotic disorders; the category “anxiety disorders” (F40-F44) included anxiety and dissociative disorders; the category “personality disorders” (F60.x) included all the personality disorder subtypes.

### Variables and measures

Information registered at first contact with the mental health services was drawn from structured fields in Electronic Health Record interface from the MEmind platform (12): sociodemographic data, coded medical and psychiatric diagnoses using ICD-10 classification. MEmind is a digital ecosystem based on an application installed on the patient’s smartphone for behavioral monitoring including self-reports, caregiver or family reports and professional assessments. The application is freely available in Apple Store and Google Play, and has been described elsewhere (13–15). Lifetime history of suicidal ideation and suicide attempts in the month prior to first contact with our psychiatric services were identified using the Columbia Suicide Severity Rating Scale (CSSRS) registered in hospitals’ Electronic Health Record (16). The CSSRS was added as a screening instrument in clinical routine from the last three years of recruitment. Data on psychiatric care costs, other medical care costs and overall healthcare costs were collected from recruitment to 31 December 2023. We used the EPICO study methodology to allocate direct costs to each care modality (including emergency room visits, hospitalization, routine laboratory testing, X Rays, computed axial tomography, magnetic nuclear resonance, psychology visits) for overall health care, psychiatric, or other medical healthcare (11). Indirect costs related to loss of productivity (cost for temporary and permanent work disability) were not computed. Each type of expense was computed over one year and expressed as mean/year (euros) per patient. Notably, psychiatric and other medical healthcare costs were calculated over different periods of time, not necessarily overlapping. Hence, each type of healthcare cost should be considered separately.

Electronic medical records were pseudoanonymized in compliance with Spanish laws on the Protection of Personal Data and guarantee of digital rights. The study was approved by the University Hospital Fundación Jiménez Díaz Ethics Committee and patients’ information was handled as stated in Spanish and European regulations on data protection and patients’ digital rights. All the participants provided written informed consent before entering the study and installing the MEmind application.

### Availability of data

Data will be made available upon request.

### Statistical analysis

All statistical analyses were performed using the Statistical Package for the Social Sciences (SPSS) version 29. First, we offered descriptive statistics of the whole population and performed univariate analyses to compare the clinical characteristics, and healthcare costs between depression subtypes. We also compared patients who had reported suicidal ideation in the previous month with those who had not, and also compared patients who had attempted suicide with those who had not recently attempted suicide. The univariate analyses were performed using F-exact test, chi-square, ANOVA or Welch’s ANOVA in case of variance inequalities assessed through Levene’s test. Effect sizes and confidence intervals (Eta-squared, [CI]) were computed (17). If the results were significant, *post-hoc* comparison between groups were applied through Tukey’s or Games-Howell’s tests. For exploratory purposes, we performed a binary logistic regression to assess the clinical factors associated with suicidal ideation and attempts.

The significance level was set at p < 0.05, using 2-sided tests and 95% confidence intervals.

## Results

### Baseline characteristics of the sample

**Figure 1:**
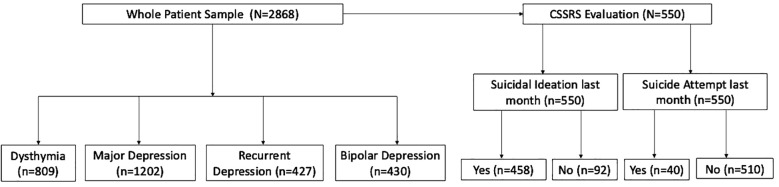
Study flow chart.

Of the 39,472 patients who received mental healthcare between 2014 and 2020, 2,868 met the inclusion criteria and were included in the analysis. Of these patients, 550 were assessed with the CSSRS at baseline, as this tool was available latter after the start of recruitment. Suicidal behavior was analyzed in this last subgroup. The study flow chart is provided in **Figure 1**.

The clinical characteristics of the study population at baseline are reported in **Table 1**. The mean age of the sample was 70.05 [SD= 7.81] and 75.9% of the patients were women (N=2177). Age classes were distributed as following: 1,639 (57.1%) were aged between 60 and 70 years; 888 (31%) were aged between 71 and 80 years; and 341 (11.9%) were aged over 80 years. Regarding marital status, 8.8% (N=246) of the patients were single (never lived with a partner and never married), 35.7% (N=994) were separated or widowed, and 55.4% (N=1542) were living with a partner. Total health care costs per patient averaged 2,811.64 euros/year [SD=22168.47].

**Table 1:** Clinical characteristics of old age patients and subsequent healthcare costs according to the clinical depression category^a^.

Clinical characteristics	Whole sample (n=2868)	Dysthymia (n= 809)	Major Depressive Disorder (n=1202)	Recurrent Depressive Disorder (n=427)	Bipolar depression (n=430)	Significance (p-value)^b^
**Age**	70.05 [7.81]	69.57 [7.08]	70.80 [8.53]	69.65 [7.27]	69.26 [7.37]	< 0.001
60-70	1639 (57.1%)	476 (58.8%)	652 (54.2%)	245 (57.4%)	266 (61.9%)	< 0.001
71-80	888 (31%)	266 (32.9%)	344 (28.6%)	145 (34%)	133 (30.9%)	
> 80	341 (11.9%)	67 (8.3%)	206 (17.1%)	37 (8.7%)	31 (7.2%)	
Gender						
Male	681 (23.7%)	65 (8.1%)	376 (31.4%)	89 (20.9%)	151 (35.1%)	< 0.001
Female	2177 (75.9%)	742 (91.9%)	820 (68.6%)	336 (79.1%)	279 (64.9%)	
Missing	10	2	6	2	–	
Family status						
Single^c^	246 (8.8%)	60 (7.6%)	99 (8.5%)	31 (7.5%)	56 (13.5%)	< 0.001
Separated or widowed	994 (35.7%)	283 (36%)	433 (37.2%)	127 (30.5%)	151 (36.4%)	
Lives with a partner	1542 (55.4%)	443 (56.4%)	633 (54.3%)	258 (62%)	208 (50.1%)	
Missing	86	23	37	11	15	
Comorbidities						
Organic mental disorder (F0)	161 (5.6%)	18 (2.2%)	78 (6.5%)	22 (5.2%)	43 (10%)	< 0.001
Substance use disorder (F1)	126 (4.4%)	26 (3.2%)	58 (4.8%)	28 (6.6%)	14 (3.3%)	< 0.001
Psychotic disorder (F2)	66 (2.3%)	10 (1.2%)	27 (2.2%)	8 (1.9%)	21 (4.9%)	< 0.001
Anxiety disorders (F4)	594 (20.7%)	249 (30.8%)	229 (19.1%)	94 (22%)	22 (5.1%)	< 0.001
Personality disorders (F6)	254 (8.9%)	127 (15.7%)	74 (6.2%)	31 (7.3%)	22 (5.1%)	< 0.001
Missing	1667	379	736	244	308	
Mean healthcare costs/year (Euros) per patient						
Total Healthcare costs (missing= 259)	2811.64 [22168.47]	1911.82 [1841.47]	2277.67 [5303.97]	1998.25 [2921.64]	7200.83 [58245.74]	0.066
Psychiatric care costs (missing= 249)	1433.68 [17530.33]	293.61 [620.04]	1903.14 [25093.83]	906.80 [2685.71]	3105.92 [19045.60]	< 0.001
Medical Healthcare costs (missing= 220)	2082.78 [11483.89]	2126.87[8638.93]	2393.20 [16323.71]	1564.49 [2360.61]	1670.56 [1955.25]	0.547

a Data are means [Standard Deviation], or number (%).

b The univariate analyses were performed using F-exact test, chi-square, or ANOVA.

c Single: never lived with a partner and never married.

In the whole sample, 28.2% (N=809) of patients were diagnosed with dysthymia, 41.9% (N=1202) with MDD, 14.9% (N=427) with recurrent depressive disorder, and 14.9% (N=430) with bipolar depression. Regarding psychiatric comorbidities, 5.6% (N=161) were diagnosed with an organic mental disorder, 4.4% (N=126) with a substance use disorder, 2.3% (N=66) with a psychotic disorder, 20.7% (N=594) with an anxiety disorder, and 8.9% (N=254) with a personality disorder. The distributions of the total, psychiatric and medical healthcare costs across the different comorbidity categories are shown in **Supplementary Table 1**.

Among patients assessed with the CSSRS (N=550), 83.2% (N=458) reported suicidal ideation, and 7.3% (N=40) had attempted suicide in the previous month. Those results are presented in the **Table 2**.

**Table 2:** Clinical characteristics of old age patients and subsequent healthcare costs according to the suicidal ideation and behaviors at baseline^a^.

Clinical characteristics	Suicidal ideation (last month)			Suicide attempt (last month)		
	Yes (n= 458)	No (n=92)	Significance (p-value)^b^	Yes (n=40 )	No (n= 510)	Significance (p-value)^b^
**Age**	68.74 [7.73]	69.63 [7.57]	0.311	72.62 [8.78]	68.59 [7.54]	0.001
60-70	296 (64.6%)	54 (58.7%)	0.550	15 (37.5%)	335 (65.7%)	0.002
71-80	113 (24.7%)	26 (28.3%)		18 (45%)	121 (23.7%)	
> 80	49 (10.7%)	12 (13%)		7 (17.5)	54 (10.6%)	
Gender						
Male	133 (29.2%)	23 (25%)	0.246	10 (25%)	146 (28.8%)	0.379
Female	322 (70.8%)	69 (75%)		30 (75%)	361 (71.2%)	
Missing	3	–		–	3	
Famlily status						
Single^c^	28 (6.2%)	7 (7.8%)	0.126	5 (12.5%)	30 (6%)	0.275
Separated or widowed	164 (36.5%)	42 (46.7%)		14 (35%)	192 (38.5%)	
Lives with a partner	257 (57.2%)	41 (45.6%)		21 (52.5%)	277 (55.5%)	
Missing	9	2		–	11	
Mood disorders						
Dysthymia	118 (25.8%)	19 (20.7%)	<0.001	5 (12.5%)	132 (25.9%)	0.025
Major depressive disorder	224 (48.9%)	36 (39.1%)		19 (47.5%)	241 (47.3%)	
Recurrent depression	85 (18.6%)	19 (20.7%)		14 (35%)	90 (17.6%)	
Bipolar depression	31 (6.8%)	18 (19.6%)		2 (5%)	47 (9.2%)	
Missing	–	–		–	–	
Comorbidities						
Organic mental disorder (F0)	19 (4.1%)	9 (9.8%)	0.030	4 (10%)	24 (4.7%)	0.138
Substance use disorder (F1)	36 (7.9%)	8 (8.7%)	0.461	2 (5%)	42 (8.2%)	0.360
Psychotic disorder (F2)	6 (1.3%)	4 (4.3%)	0.069	2 (5%)	8 (1.6%)	0.160
Anxiety disorders (F4)	139 (30.3%)	14 (15.2%)	0.002	12 (30%)	141 (27.6%)	0.437
Personality disorders (F6)	67 (14.6%)	17 (18.5%)	0.215	6 (15%)	78 (15.3%)	0.588
Missing	–	–		–	–	
Suicidal behavior lifetime						
No	335 (73.1%)	3 (3.3%)	<0.001	7 (17.5%)	331 (64.9%)	<0.001
Yes	123 (26.9%)	89 (96.7%)		33 (82.5%)	179 (35.1%)	
Mean healthcare costs / year (Euros) per patient						
Total Healthcare costs	2366.15 [3270.35]	3412.55 [5407.00]	0.017	3559.46 [3530.69]	2468.22 [3746.87]	0.091
Psychiatric care costs	2187.39 [23193.70]	1413.11 [4199.10]	0.755	4075.86 [9195.10]	1905.56 [21833.88]	0.554
Medical Healthcare costs	1921.42 [2714.51]	2545.63 [4557.53]	0.144	2037. 82 [1522.81]	2009.27 [3185.63]	0.959

a Data are means [Standard Deviation], or number (%).

b The univariate analyses were performed using F-exact test, chi-square, or ANOVA.

c Single: never lived with a partner and never married.

### Association between depression subtypes, and patients’ characteristics or healthcare costs over the follow-up

Age differed between depression subtypes (Eta-squared 0.007 CI [0.002 - 0.013]; p<0.001). Those diagnosed with MDD were older than those with dysthymia, recurrent depressive disorder or bipolar depression (Mean 70.80 [8.53] Vs. 69.57 [7.08] (p=0.002), 69.65 [7.27] (p=0.038), and 69.26 [7.37] (p=0.002), respectively). Particularly, 17% of the patients with MDD were aged over 80 years, while they were 8.3% in the group showing dysthymia, 8.7% in the group with recurrent depressive disorder, and 7.2% in the group with bipolar depression. In contrast, patients with bipolar depression were younger than the other groups, 61.9% of them being aged between 60 and 70 years old. Those diagnosed with dysthymia were more frequently females than those with MDD, recurrent depressive disorder or bipolar depression (91.9% vs. 68.6%, 79.1%, and 64.9%; p<0.001 respectively).

Patients with bipolar depression lived less frequently with a partner (50.1%) than other groups (56.4% for dysthymia, 54.3% for MDD and 62% for recurrent depressive disorder, p<0.001). They also had more frequent comorbidities with organic mental disorders (10%) and psychotic disorders (4.9%).

Those with recurrent depressive disorder had a higher comorbidity rate with substance use disorder (6.6%). Dysthymia was more frequently comorbid with anxiety disorder (30.8%) and personality disorder (15.7%).

**Figure 2:**
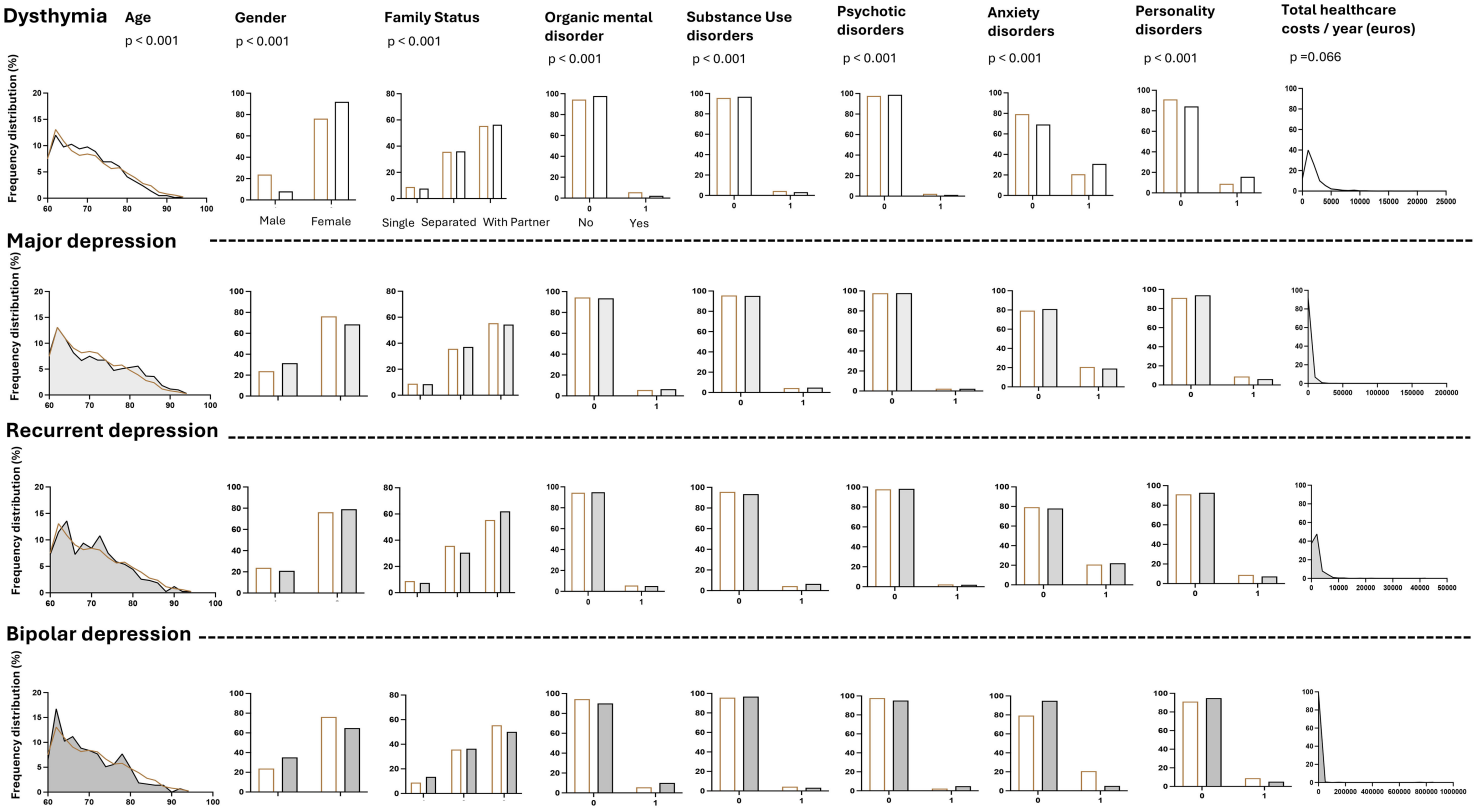
Clinical characteristics of old age patients and subsequent total healthcare costs/year (euros) according to the clinical depression category. The category “organic mental disorders” (F00-F09) includes Alzheimer’s dementia, delirium and other dementia; the category “substance use disorders” (F10-F19) includes all the mental and behavioral disorders due to psychoactive substance use; the category “psychotic disorders” (F20-F25, F28) includes schizophrenia, delusional disorders and other nonorganic psychotic disorders; the category “anxiety disorders” (F40-F44) includes anxiety and dissociative disorders; the category “personality disorders” (F60.x) includes all the personality disorder subtypes. Single: never lived with a partner and never married.

Finally, psychiatric healthcare costs over the follow-up differed between the depression subtypes (Eta-squared 0.003 CI [0.000 - 0.008]; p<0.001). They were significantly higher in patients diagnosed with bipolar depression (Mean 3105.92 [SD=19045.60] euros/year), than in those diagnosed with dysthymia (Mean 293.61 [SD=620.04] euros/year, p=0.026), but did not differ from those bearing MDD (Mean 1903.14 [SD=25093.83] euros/year, p= 0.775) or recurrent depressive disorder (Mean 906.80 [SD=2685.71] euros/year, p= 0.129). The results are shown in **Table 1** and in **Figure 2**.

### Association between suicidal ideation, and patients’ characteristics or healthcare costs over the follow-up

Patients who reported suicidal ideation in the previous month were more frequently diagnosed with dysthymia and MDD, and less frequently diagnosed with recurrent depressive disorder and bipolar depression compared with those without suicidal ideation (p<0.001). The individuals with suicidal ideation in the previous month had less frequent comorbid organic mental disorder (p=0.030) and more frequent anxiety disorders (p=0.002). They were also less likely to report a history of suicide attempts (p<0.001). Finally, total healthcare costs were reduced in patients with suicidal ideation in the previous month (p=0.017). The results are reported in **Table 2**.

### Association between suicidal attempts in the last month, and patients’ characteristics or healthcare costs over the follow-up

**Figure 3:**
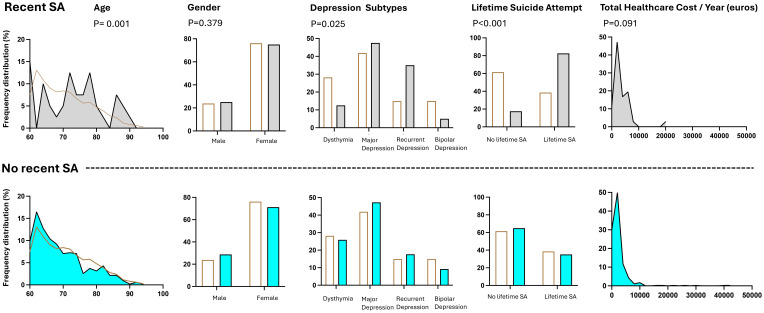
Clinical characteristics of old age patients and subsequent healthcare costs according to the suicidal ideation and behaviors at baseline.

Patients who attempted suicide in the month prior to evaluation were older (p=0.001), more frequently diagnosed with MDD and recurrent depressive disorder, less frequently diagnosed with dysthymia and bipolar depression (p=0.025), and showed more frequent lifetime history of suicide attempts (p<0.001) than those who did not attempt suicide. No differences were found regarding the healthcare costs over the follow-up. The results are reported in **Table 2** and **Figure 3**.

### Exploration of the factors associated with suicidal ideations and attempts using multivariate models

In the multivariate analysis, the frequency of suicidal ideation was increased in participants showing a comorbid anxiety disorder (OR = 2.113; CI-95% 1.027-4.346). In the contrary, suicidal ideation was less frequent in individuals reporting a lifetime history of suicide attempt (OR= 0.013; CI-95% 0.004-0.044). These latter results contrasted with what was found for suicide attempts. In fact, recent suicide attempters had a more frequent lifetime history of suicide attempt (OR= 8.434; CI-95% 3.491-20.372). Recent suicide attempts were also more frequent in age ranges between 71 and 80 years (OR= 3.433; CI-95% 1.476-7.988), and over 80 years (OR= 3.322; CI-95% 1.141-9.667) when compared to the individuals aged between 60 and 70 years. Suicide attempts in the previous month were also more frequent in patients diagnosed with recurrent depressive disorders (OR= 3.529; CI-95% 1.105-11.266). The results are shown in the **Table 3**.

**Table 3:** Association between clinical characteristics and suicidal ideation or behaviors at baseline^a^.

Clinical characteristics	Suicidal ideation			Suicide attempt (last month)		
	Estimated odds ratio (exp(b))	95% CI for Exp(b)	Significance (p-value)	Estimated odds ratio (exp(b))	95% CI for Exp(b)	Significance (p-value)
Age						
60-70						
71-80	0.878	0.447-1.728	0.707	3.433	1.476-7.988	0.004
> 80	0.833	0.326-2.130	0.703	3.322	1.141-9.667	0.028
**Gender (female)**	0.689	0.344-1.380	0.293	1.956	0.772-4.959	0.157
Mood disorders						
Dysthymia						
Major depressive disorder	1.238	0.556-2.753	0.601	2.452	0.785-7.658	0.123
Recurrent depression	1.094	0.459-2.611	0.839	3.529	1.105-11.266	0.033
Bipolar depression	0.570	0.210-1.549	0.271	0.362	0.038-3.479	0.379
**Organic mental disorder (F0)**	0.501	0.151-1.665	0.260	1.720	0.472-6.273	0.411
**Anxiety disorders (F4)**	2.113	1.027-4.346	0.042	1.776	0.766-4.119	0.181
**Suicidal behavior lifetime**	0.013	0.004-0.044	<0.001	8.434	3.491-20.372	<0.001
**Total Healthcare costs / year per patient (> Median, Euros)**	0.753	0.405-1.403	0.372	1.534	0.659-3.568	0.321

a A binary logistic regression was performed to assess the clinical factors associated with suicidal ideation and attempts.

## Discussion

In this naturalistic cohort study, we found that the elderly with MDD were older than those with other clinical depression subtypes, whereas those with bipolar depression were younger, were more often socially isolated and showed more frequent mental organic disorders and psychotic comorbidities. Older adults with bipolar depression also required more psychiatric healthcare resources than those diagnosed with dysthymia, while the latter effect remains small.

We also found that older age, a diagnosis of recurrent depression and a lifetime history of suicide attempts increased the risk of attempting suicide. Suicidal ideation was more frequent in the participants showing comorbid anxiety disorder. In contrast, a lifetime history of suicidal behaviors was related with a lower risk of recent suicidal ideation. In our sample, suicidal ideators or attempters did not use higher healthcare resources than non-suicidal individuals.

### Patients’ profiles differ between clinical depression subtypes in old age

To our knowledge, no studies have previously compared patient characteristics between clinical subtypes of depression systematically in old age. However, our results are in line with some indirect findings in the literature. In particular, it has been reported that female gender and younger age may be associated with a higher risk of depression recurrence (18). Indeed, recurrent depression may be detected earlier as affected individuals experience multiple episodes in younger age (8). Similarly, in our study the individuals diagnosed with recurrent depressive disorders were more frequently females and were younger than those diagnosed with MDD, while age differences across depression subtypes were of small magnitude and showed limited clinical significance.

Furthermore, younger age at onset has been reported in the elderly with bipolar depression compared with those showing unipolar MDD (19). Earlier onset of bipolar depression has also been reported in middle-aged populations (20, 21). In addition, late-onset bipolar disorder was more frequently of organic origin (22). Bipolar depression shows particularly high burden of comorbidities, whether psychiatric or organic (23). In line with the literature, we evidenced that the elderly showing bipolar depression were mostly younger, were most often socially isolated and were most frequently diagnosed with organic mental disorder and psychotic comorbidities.

### Specificities of suicide attempter’s profile

A recent review highlighted that despite a large amount of studies evaluating the effect of age, its role as a risk factor for suicide attempt was contrasted (24). In our sample, depressed elderly aged over 70 had an increased risk to attempt suicide compared with those aged between 60 and 70. In the same line, the risk was higher in those aged over 80 years compared to those aged between 60 and 70 years, while the effect was weaker. Furthermore, previous studies evidenced that the recurrence of depression was associated with a higher likelihood to attempt suicide (25). Our results support those findings as the diagnosis of recurrent depression was the only mood disorder increasing the risk to attempt suicide in the elderly. Conversely to the elevation of the risk to attempt suicide reported in the middle age population with bipolar disorder (26), bipolar depression in the older adults was not independently associated with such higher risks. In addition, we found that a history of suicidal behavior was the strongest risk factor for suicide attempts in later life, with an 8-fold increased risk. In their systematic review, Beghi et al. (2021) reported a moderate role of the lifetime history of suicidal behaviors in the elderly, with only 4 upon 9 studies showing significant results (24). In their meta-analyses grouping longitudinal studies performed in adolescents and adults, Ribeiro et al. (2016) found that self-injurious thoughts and behaviors were linked with an increased risk of attempting suicide (OR of 2.16), while a history of suicide attempt was specifically associated with increased odds of suicide attempt (OR 3.61) over the follow-up. The association did not differ between age groups (27). The latter results were partly obtained in general population samples. In our study, the data were collected in a clinical population seeking hospital mental care, and the suicidal vulnerability may have cumulated with the effect of current mood disorder at inclusion, thus increasing the likelihood of suicidal acts.

Surprisingly, we found that previous suicide attempts were associated with a lower risk of suicidal ideation in later life. Those results diverged from previous findings in the general population. In particular Ribeiro et al. (2016) reported a slight but significant positive association between the occurrence of suicide attempt and subsequent suicidal thoughts (OR 1.58) in their meta-analysis (27). Interestingly, a recent meta-analysis showed that while prior self-injurious thoughts and behaviors increased the risk for subsequent suicidal ideation in the general population, the association lost significance among veterans (28). Authors indicate that the relationship between risk factors and suicide related outcomes may weaken with age. This outlines the specificities and differences between the mechanisms underlying suicidal behaviors and ideation across the lifespan. The relationship between a preexisting vulnerability to suicide and subsequent suicidal ideation in older adults may vary from the one observed in younger individuals. However, further studies should be conducted to replicate these findings and confirm this hypothesis. In addition, comorbid anxiety was a risk factor for suicidal ideation in our study. These results are in line with prior study from Bendixen et al. (2018) conducted in aged individuals (29). This relationship seems stronger in elderly than in younger adults (30). This points the need toward treating such anxiety symptoms for preventing suicidal ideation in late life.

Finally, we did not evidence any relationship between gender and suicidal ideation nor with suicidal attempts. Similarly, absence of relationship was shown in a community sample of individuals bearing borderline personality disorders (31). However, in a population of patients with bipolar disorders, females were more likely to attempt suicide (32). In the older adults, rates of suicidal ideation and attempts differed between genders (33). Suicide deaths were found to be up to six time higher in males than in females (34). Hence, further studies should investigate and precise gender differences toward suicidality in the elderly population.

### Healthcare associated costs

Previous Spanish real-world research evaluated the medical costs in retrospective observational studies involving depressed people, and showed that they ranged from a mean of 3,402€/patient/year for non-TRD patients (11), to 3,846 €/patient/year (35). In our study sample, the mean total healthcare costs averaged 2811 €/patient/year. However, those costs were underestimated as we only calculated direct expenses. In fact, the studies conducted by Vieta (2021) and Perez-Sola (2021) also included indirect costs related to temporary and permanent disability. Annual psychiatric healthcare expenses in elderly patients diagnosed with bipolar depression exceeded those with dysthymia in our sample, but also out passed the total healthcare costs described in the above studies. These results are in line with previous reports showing that elderly with bipolar depression were four times more likely to use mental health services and four times more likely to require hospitalization (36). Nevertheless, interpretation should be cautious as measuring the likelihood to use healthcare resource is not strictly equivalent with the costs associated with healthcare. Furthermore, the large variability of the total healthcare costs in the patients with bipolar depression may have prevented us to evidence differences with other depression subtypes. Regarding the impact of suicidal behavior, although healthcare costs following a suicide attempt seemed more elevated, there was no significant difference compared with non-attempters.

### Limitations and strengths of the study

Our study has some limitations. First, we were not able to distinguish between late-onset and early-onset depressive disorders in our elderly sample. In fact, reliable information regarding eventual past depressive episodes in middle or young age was not available. Furthermore, data on clinical factors classically associated with suicidal attempts were lacking, such as cigarette smoking or impulsivity traits (37). Data collection period overlapped with SARS-CoV-2 pandemic. This may limit the generalizability of the results. Moreover, while we assessed the presence of organic mental disorders including dementia, data collection did not involve recordings of cognitive measures. In fact, altered cognitive function may be associated with depression subtypes and suicidal behaviors. Regarding the calculation of medical expenses, mean total, medical, and psychiatric costs per year were computed over different time periods. Hence, each type of healthcare costs should be considered separately. Finally, the calculated costs did not include indirect social costs related with patient disability or premature mortality.

However, such knowledge may help guide healthcare policies for the prevention of depressive disorders in the elderly, and may help target clinical subgroups with particular needs. Notably, our research is a naturalistic real-world study involving one of the largest samples of depressed older adults to date. As we focused on a population receiving specific hospital psychiatric care, our findings may be generalized to frail older people, who tend to have the worst medical outcomes.

### Clinical implications

Our findings have clinical implications. Early preventive measures should focus on detecting and treating comorbid anxiety in older adults in order to reduce the occurrence of suicidal ideation. In addition, tailored and specific treatments for recurrent depression should be provided early to help prevent suicidal behaviors. To this end, the use of innovative technologies for detecting and treating these conditions should be encouraged. For instance, smartphone-based tools and digital monitoring show promise for the care of older patients, particularly in populations with limited resources or those who are socially isolated (38).

### Conclusions

Overall, we highlighted the phenotypes specifically associated with clinical depression subtypes across the depression spectrum in older age. We also examined the characteristics associated with suicidal behavior in older adults seeking mental healthcare in outpatients and inpatient hospital settings. Among the depression categories assessed, elderly individuals with bipolar depression were the youngest, most frequently socially isolated, and exhibited the highest rates of mental organic disorders and psychotic comorbidities. Psychiatric healthcare costs differed between depression subtypes, with a small effect. Early secondary and tertiary preventive measures should be set in order to mitigate the medical impacts of comorbidities. In parallel, being older, being diagnosed with recurrent depression, and showing a history of suicidal attempts increased suicide risk, while the latter factor was conversely associated with suicidal ideation. Clinicians should particularly assess eventual suicidal behaviors, preparation and plans in those patients for efficient prevention. To this end, the use of innovative technologies for detecting the behavioral markers of suicide in the elderly should be encouraged.

## Data Availability

The raw data supporting the conclusions of this article will be made available by the authors, without undue reservation.

## Memind group collaborators

María Luisa Barrigón, Jorge Lopez-Castroman, Phillippe Courtet, Antonio Artés-Rodriguez, Enrique Baca-Garcia, Fuensanta Aroca Bisquert, Ana De Granda-Beltrán, José Luis Palomo, Elizabeth Madridejos, Lucía Albarracín García, Carmen Aguilar Castillo, Laura Jiménez-Muñoz, Sofía Abascal-Peiró, Ana Pérez-Balaguer.
