# Supplementary material for: Depression subtypes, suicidality, and healthcare costs in older adults: results from a naturalistic study
Source: Front Psychiatry. 2025 Jun 17;16:1560719. doi: 10.3389/fpsyt.2025.1560719 (PMC12209252; doi:10.3389/fpsyt.2025.1560719)
Supplement: Supplementary file 1 [file Table1.docx]

| Mean costs / year (Euros) | Organic mental disorder (n=161) | Substance use disorder (n=126) | | Psychotic disorder (n=66) | Anxiety disorders (n=594) | Personality disorders (n=254) |
| --- | --- | --- | --- | --- | --- | --- |
| Total Healthcare costs | 2755.66 [3399.27] | 2233.97 [1994.98] | 3403.51 [3773.25] | | 2051.51 [2340.01] | 2041.91 [1716.94] |
| Psychiatric care costs | 7337.36 [57493.65] | 646.68 [961.72] | 2649.26 [4549.98] | | 1368.86 [20268.16] | 613.31 [1104.25] |
| Medical Healthcare costs | 1972.97 [1770.57] | 2026.37 [1948.93] | 1491.49 [1372.62] | | 1858.20 [2171.47] | 1693.10 [1478.34] |
